# Is empathy one of the Big Three? Identifying its role in a dual-process model of ideology and blatant and subtle prejudice

**DOI:** 10.1371/journal.pone.0195470

**Published:** 2018-04-05

**Authors:** José Luis Álvarez-Castillo, Gemma Fernández-Caminero, Hugo González-González

**Affiliations:** Department of Education, Faculty of Education Sciences, University of Cordoba, Cordoba, Spain; University of Bologna, ITALY

## Abstract

In the field of the social psychology of prejudice, John Duckitt's Dual-Process Cognitive-Motivational Model of Ideology and Prejudice has gained a firm grounding over the past decade and a half, while empathy has become one of the most powerful predictors of prejudice, alongside right-wing authoritarianism and social dominance orientation. This study integrates empathy into the dual-process model, exploring the effects of this variable, along with the impact of personality and ideological attitudes, on prejudice in both its blatant and subtle forms. A cross-sectional research design was used to collect data from 260 university students by self-report measures. Despite its cross-sectional nature, a pattern of causal relationships was hypothesized according to experimental and longitudinal findings from previous studies. The path analysis results show that in the model fitted to the data, empathy does not have any direct impact on prejudice, although it plays a significant role in the prediction of prejudice towards a particular immigrant group. On the other hand, the dual-process model is confirmed in the explanation of blatant prejudice and, in a weaker and indirect way, of subtle prejudice; sustaining the distinctive nature of these constructs on some differential predictors and paths. In the discussion, this study proposes that when ideological and personality-based variables are both included in the model, general empathy is not so robust in the explanation of prejudice, since some of the empathetic components might become diluted among other covariates. But even so, its indirect effectiveness through personality and ideological attitudes remains relevant.

## Introduction

In an interdependent world, where we interact with each other in increasingly diverse physical and virtual spaces, stereotypes and prejudices form the basis of attributions, emotional reactions and daily behaviours presumably with greater frequently than in the past. However, people who apply these constructs generally do so in an indirect and subtle way in order to avoid conflict or protect themselves or their group in the pursuit of their goals. The ongoing social relevance of these interpersonal biases continues to encourage research into this phenomenon, since the publication of Allport's seminal study [1]. For example, on the Web of Science there are 902 social science publications from the period 1983–1999 with the term "prejudice" in the title. Then, over the following 17 years (2000–2016), this figure rose to 2,232. In a more advanced search, using the sequence "prejudice or stereotyp* or discriminat* or racis* or sexis* or homophob*", the number of publications in these two periods increases to 15,608 and 28,562, respectively. Therefore, interest in research on intergroup biases, as seen in the increased publication of studies in quality sources, is clearly still on the rise.

Obviously, this is not just about documenting quantitative development, but the creation of models that have been steadily gaining firm theoretical grounding. Some of these theoretical developments have adopted general empathy, or some of its various components, as one of their most stables bases. However, although empathy's direct effect on prejudice and its mediational role in simple models is already well documented, there is still no wider approach that covers the effectivity of both personality and ideological attitudes on prejudice, through empathetic mediation in dual-explanation approaches. This study represents progress in this field by exploring the pathways of the effects of three personality traits, attitudes of authoritarianism and social dominance, and empathy on prejudice, in both its blatant and subtle forms. It aims therefore to continue the qualitative development of research in the field of the social psychology of prejudice, making progress in terms of models that are more complex and, at the same time, more parsimonious.

### Personality, ideological attitudes and prejudice

In many studies, agreeableness and openness to experience feature as two of the Big Five factors that are very closely linked to prejudice [2–7]. Agreeableness, as the opposite of antagonism, includes components such as tender-mindedness and altruism, as well as compliance and straightforwardness, which are negatively associated with prejudice. Similarly, openness covers facets such as non-conformity and unconventionalism; it is inversely linked to authoritarianism and positively related to liberal socio-political values. These components explain, for example, that people who are agreeable and open to experience tend to have more favourable attitudes towards immigration [3] and are more tolerant of social diversity [8].

These personality traits also play a key part in John Duckitt's Dual-Process Cognitive-Motivational Model of Ideology and Prejudice [9–12]. This theoretical approach predicts that the trait of openness to experience negatively anticipates right-wing authoritarianism (RWA). This is confirmed in the meta-analysis performed by Sibley and Duckitt [12], based on 71 studies involving 22,068 participates (see also [13–15]). Authoritarianism, in turn, would have a significant explanatory effect on prejudice. Altemeyer [16] conceptualised the various components of authoritarianism, defining authoritarian aggression as a general aggressiveness directed at groups seen as punishable by the competent authority; authoritarian submission as compliance with the designs of a legitimately established authority in society; and conventionalism as adhesion to the social norms that society and the competent authorities are seen to assume. Considering these semantic ingredients, openness to experience would be a disposition that, at low levels, helps form a view of the world as an unsafe and dangerous place, and the goal of maintaining social order and security, determining prejudice towards threatening groups.

On the other hand, agreeableness would be the trait that, at low levels, predicts social dominance orientation (SDO) which, in turn, predicts prejudice towards groups with lower status and power [12]. In the words of Sidanius and Pratto [17], SDO refers to a "very general individual differences orientation expressing the value that people place on nonegalitarian and hierarchically structured relationships among social groups" (p. 61).

This differential effect of personality on attitudes has been confirmed by Sibley and Duckitt [18] and by Perry and Sibley [13] in longitudinal cross-lagged panel designs of 1 year and nine months, respectively. In this model, the direct impact of personality on prejudice is weak; however, its indirect impact through attitudes (RWA and SDO) is much stronger [10–12,19]. This is also found in the third study by McFarland [20], in which, despite the significant correlations of four of the Big Five with prejudice, none of these traits added additional variance in the direct prediction of prejudice through hierarchical regression analysis. As such, research under the framework of the dual-process model differs from that carried out under assumptions of prejudice being directly predicted by personality, as suggested by Ekehammar and Akrami [4,5]. However, Bo Ekehammar himself has found in various studies how well his data fits to causal models in which personality influences different types of prejudice through RWA and SDO [21,22].

In fact, more recently, Bergh and Akrami [23] found weak evidence of the link between agreeableness and prejudice, when this trait was analysed in conjunction with honesty-humility and the facet of altruism from the HEXACO model, which showed negative effects on prejudice. On the other hand, the definition of altruism overlaps with the concept of empathy, placing empathy among the facets of personality measured by the HEXACO model. Likewise, Bergh, Akrami, Sidanius and Sibley [24] found with sample groups from three different countries (USA, Sweden and New Zealand) that only some facets of agreeableness predict prejudice, while the HEXACO model attributes are stronger predictors (honesty-humility and altruism). In turn, in the model of the Big Five Factors, openness would retain its power of prediction.

All these studies used different measures of prejudice; some using instruments to measure classic prejudice (e.g. [2]), others measuring subtle or modern prejudice (e.g. [7,22–25]) and some making use of both techniques (e.g. [4,5]). Although direct, blatant or classic prejudice shows consistent correlation with indirect, modern, subtle or symbolic prejudice [4,26], these are in fact different intergroup biases [27,28]. As such, it would be interesting to determine if the pattern of associations between personality, ideological attitudes and prejudice differs depending on the specific type of prejudice, i.e. classic or modern. There have been very few studies in this respect. One of them was conducted by Passini and Morselli [29], who found that SDO had a direct effect on blatant prejudice, but not on subtle prejudice, which is fully mediated by moral inclusion (an institutionalised and covert form of inclusion). In a previous study, Van Hiel and Mervielde [30] had found that the correlation between SDO and subtle prejudice does not reach significance when RWA is partialled out. On the other hand, RWA might behave in a similar way with both types of prejudice [30,31], although some results support its higher predictive power on blatant prejudice [32]. Nevertheless, more in-depth knowledge is required about this differential status in dual-process models.

### Empathy: Concept and prosocial dimension

The literature shows considerable diversity with regard to the definition of empathy as a construct [33–35]. However, by way of introduction, it could be understood as an emotional and vicarious type of experience about the feelings of another human being [36]. Nevertheless, revised research suggests that this concept could be approached in many different ways and has multiple components that, although similar and in some cases complementary, are of interest in various fields: psychology, sociology, intercultural studies, pathology, gender studies, religious studies, neurology, etc. [34,35,37–43]. Despite these diverse approaches, certain consensus has been reached about the different components of empathy and some of its behavioural counterparts. As such, empathy could be said to have three basic components: affective sharing, relating to the ability to respond to the emotions of others; empathic concern, associated with the desire to care for the other; and perspective taking, referring to the ability to put oneself in the other's place and imagine what they think and feel [44].

With regard to the covariables of empathy, its importance in people's prosocial disposition has come to light in recent years [8,45–53], along with its positive role in conflict resolution [37,38,54–56], and it is now seen as one of the key motivations of personality and altruistic values [57,58]. In relation to this framework, Eisenberg [59] highlighted the role of empathy in moral development and conceptualised it as a reaction originating in an understanding of the situation of others, orientated towards experiencing their feelings. That is, it is about understanding other people and taking their perspective, based on both the individual information observed and information recovered from memory. In addition, empathy produces an affective response that consists in sharing the emotional state of others; i.e. feeling sadness, distress and other emotions. It also triggers behaviour that is as morally significant as it is prosocial. On the other hand, Pohling, Bzdok, Eigenstetter, Stumpf and Strobel [60] used mediation analysis to verify the significant effects of the affective dimension of empathy (empathic concern) on ethical competence through the mediating effect of various values (benevolence, conformity, tradition, power and hedonism). However, the relationship between empathy and morality was found to be complex, to the extent that the findings only supported empathetic capacity having a moderate role in moral decision-making processes. Cognitive reasoning, for example, could have an equally important effect on justice and morality in decision-making processes [44].

If we then add empathy's link with stereotyping and prejudice; social exclusion; and explicit or implicit intergroup attitudes [61–69], the argument becomes so much clearer for championing research and practices concerning the use of empathetic strategies in multi-cultural educational environments [45,70,71], or the use of different intercultural education programs to develop empathy [72–74]. In other words, empathy has been included as a component of anti-bias education in order to improve affiliative sociocultural relationships and attitudes.

### Empathy, personality and ideological attitudes

In addition to the relationship between empathy and the above-mentioned variables, empathy also has a close and important connection with personality. Del Barrio, Aluja and García [75] established over a decade ago that the link between empathy and personality factors (the Big Five) had not been sufficiently studied. Their study, involving a group of 832 Spanish adolescents, found a strong link between empathy and agreeableness (see also [76]). This research problem has been repeatedly addressed in recent years. In fact, according to Habashi, Graziano and Hoover [77], it is now obvious that prosocial processes, including emotions, cognition and behaviour, can be part of a broader motivational process linked to personality.

The findings of Magalhães, Costa and Costa [78], for example, confirmed empathy's positive links to agreeableness and openness, in a study on university medical students. Their findings led them to suggest that the personality of students be taken into account to promote greater empathy in degree programs (see also [79]). In a study on a similar sample group, Song and Shi [80] also found links between these personality traits and empathy; however, they discovered these links by differentiating between three dimensions of empathy: perspective taking, empathic concern and personal distress. As such, agreeableness was found to strongly predict empathic concern and, in a more moderate fashion, perspective taking. In turn, openness had a slight association with perspective taking and personal distress (with the latter, the regression coefficient adopted a negative valence); and neuroticism strongly predicted personal distress and was weakly associated with perspective taking. Lastly, consciousness only slightly predicted perspective taking. Overall, in this study, empathy appears more connected to agreeableness and neuroticism than to openness to experience. Melchers et al. [81] also found differential effect sizes in the association between the Big Five personality traits and empathy, in groups of university students in four countries (China, USA, Germany and Spain). These authors used two instruments to measure empathy. In the unidimensional Empathy Quotient, they detected, in order of importance, agreeableness, consciousness and openness to experience as the most important predictors. While in the Interpersonal Reactivity Index, a multidimensional instrument, agreeableness appeared as the most explanatory predictor of cognitive and affective empathy, while followed by openness, but only as a predictor of perspective taking (cognitive dimension of empathy). Lastly, neuroticism was identified as the best predictor of personal distress.

Empathy has also been attributed a mediational role between personality and different types of intergroup attitudes. For example, Butrus and Witenberg [8] identified empathic concern as the most powerful predictor of behavioural dimensions of tolerance. However, this component of empathy simultaneously appeared to take on a mediational role between openness and agreeableness, and tolerance. As such, empathy would be a more immediate predictor of tolerance, while the personality traits associated with tolerance would predict it in a more mediate fashion.

In turn, ideological attitudes, whose mediation between personality and prejudice is the most solid pillar of Duckitt's dual-process model [9–12], are also associated with empathy. In the case of SDO, Sidanius and his colleagues [82] confirmed its relationship with empathy in a longitudinal cross-lagged panel design. These authors measured two affective components of empathy–empathic concern and compassion–, showing both as causes of SDO, but also as effects. The negative association between empathy and SDO has already been found in previous studies with other designs [20,83]. In a study by McFarland [20], the effect of SDO on empathy was unidirectional (more intense among adult participants and more moderate among students), finding the direct effect of empathy on prejudice to be of a light intensity in the adult group and a moderate intensity in the student group. In the path analysis performed by Bäckström and Bjöeklund [83], empathy's direct impact on prejudice was of a light intensity, while it had a much stronger effect on SDO which, in turn, had a heightened effect on prejudice. In the field of neuroscience, the relationship between these constructs is also being researched, but this work is still in the early stages [84].

In a similar vein, RWA is also negatively associated with empathy. Recently, Onraet, Van Hiel, De Keersmaecker and Fontaine [85], in a study on the link between emotional intelligence, ideological attitudes and prejudice, found one component of empathy (perspective taking) to have a direct effect on authoritarianism, yet no significant effect was detected from a second component (empathic concern). Nevertheless, both dimensions of empathy had an impact on SDO. A decade before, in a group of adults studied by Bäckström and Bjöeklund [83], empathy was already found to have a considerably smaller effect on RWA than on SDO. In fact, McFarland [20] found no evidence of any effect between RWA and empathy in his structural equation model. Even with these findings, consistent conclusions can still not be reached regarding the predictive effects between these two constructs.

### The present study

After reviewing the relationships between personality, ideological attitudes, empathy and prejudice, a study was envisaged that would allow us to explain this latter variable based on personality (agreeableness, openness and neuroticism), ideological attitudes (right-wing authoritarianism and social dominance orientation) and general empathy, while also considering possible variations depending on the empathetic components. Although John Duckitt's model [9–12] has established a dual-process approach to explaining prejudice, as described in the theory review, empathy has not yet been considered in this model, despite evidence of the relationship between this variable and personality and separately with ideological attitudes. What is now needed is to confirm whether empathy plays a mediational role while at the same time having a direct impact, albeit weaker, on prejudice when introduced into the above-mentioned model.

**Fig 1:**
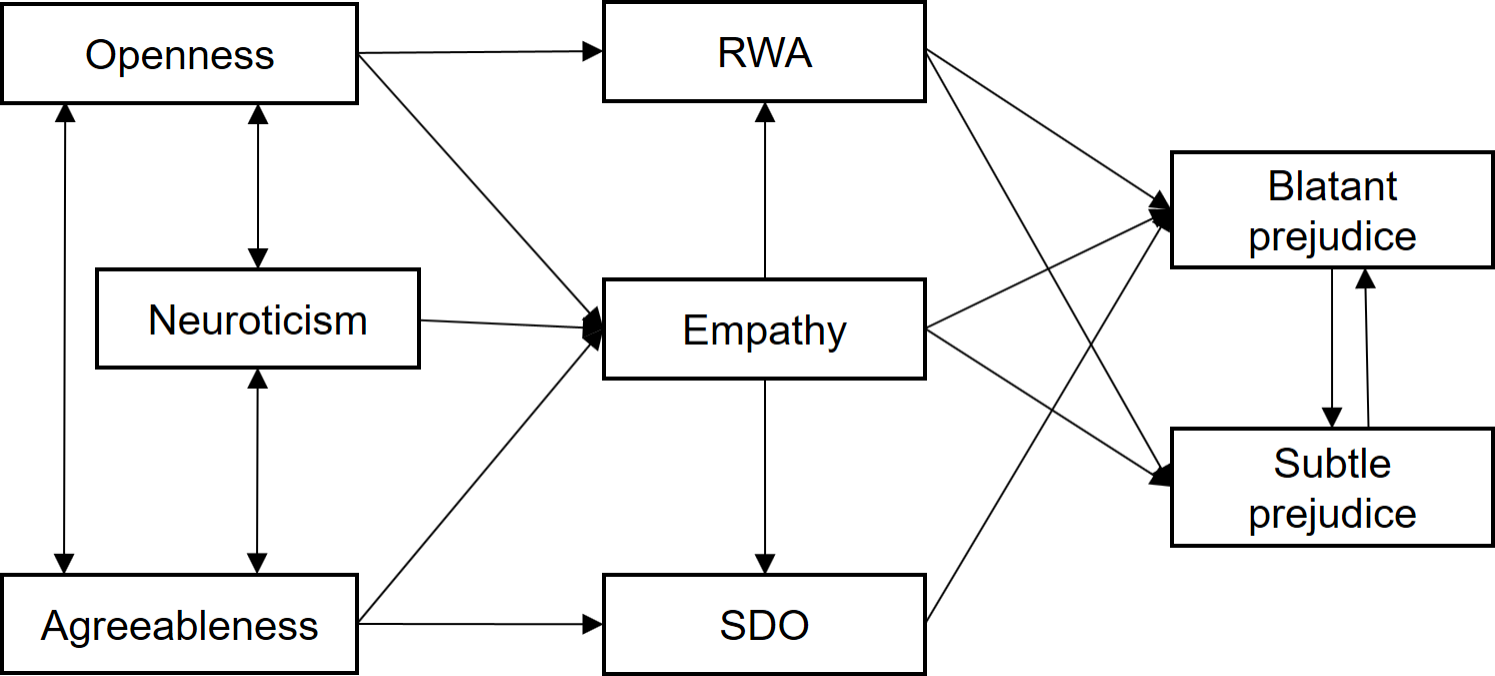
Hypothetical model on the impact of personality, attitudes and empathy on blatant and subtle prejudice.

This study also aims to test whether the explanatory dual pathway of the model can be confirmed, after introducing empathy, in the explanation of both blatant and subtle prejudice. Given that some variable predictors might have a stronger association with blatant prejudice than with subtle prejudice, the hypothesis was that when both types of bias were included in the model, blatant prejudice would receive the full effects anticipated in the dual-process theory, masking some of the weaker effects on subtle prejudice that could be exercised, when applicable, by personality and ideology. These expectations are presented together in Fig 1.

## Method

### Design

This was a questionnaire-based, cross-sectional study aimed at explaining the relationships presented in Fig 1 through confirmation using path analysis. Despite the cross-sectional nature of the study, it tested whether the set of effects fitted the expected findings under the assumption of a pattern of causal relationships, about which there is ample past evidence in the Dual-Process Cognitive-Motivational Model, obtained through experimentation and longitudinal cross-lagged panel design [13,18,19,82,86,87].

### Participants

260 university students on undergraduate education courses at the University of Cordoba (Spain) participated voluntarily in the study (*M*_*age*_ = 20.5, *SD* = 2.84; 82.3% female). They were recruited by their teachers and given extra credit in their courses for their participation.

### Variables and instruments

#### Personality

Three of the Big Five traits were measured: Openness to Experience, Agreeableness and Neuroticism; using 36 of the 60 items in the Neo-Five Factor Inventory (Neo-FFI), which is a pared-down version of the NEO Personality Inventory-R (NEO-PI-R) [88]. The Spanish version used in this study was that adapted by Cordero, Pamos and Seisdedos, published by TEA in its second edition [89]. Cronbach's alpha of .82 was obtained for Openness to Experience, .83 for Agreeableness and .90 for Neuroticism. In our data, these values were initially 73, .67 and .86, respectively. Given that internal consistency did not reach the .70 threshold in Agreeableness, the quality of each of the items comprising this factor was revised, identifying one ("If I don't like someone, I let them know") whose correlation with the factor total was practically null (*r* = -.02). With this item supressed, the consistency of the factor rose to .71. Therefore, this element was omitted in the critical analysis, and the individual scoring for Agreeableness was calculated based on the 11 remaining items. A five-point response scale was used (1 = “Totally disagree”, and 5 = “Totally agree”). Nineteen of the thirty-six items were reverse coded (seven from Openness, eight from Agreeableness and four from Neuroticism).

#### Right-wing authoritarianism

The Right-Wing Authoritarianism Scale, developed by Altemeyer [90], was selected to measure this variable for its excellent psychometric quality [9], adapted into Spanish by Seoane and Garzón [91]. Magallares [92] reports an alpha of .83 and in our study, a similar value was recorded: .82. Participants were asked to respond to the 30 items of the version using a scale of one to five (1 = “Totally disagree”, and 5 = “Totally agree”). This format was used to help participants attribute meaning to each point of the scale across the full set of measures used in the study. Half the items included statements that were reverse coded (2, 4, 7, 8, 10, 11, 13, 15, 18, 20, 21, 24, 25, 27, 29).

#### Social dominance orientation

The Social Dominance Orientation Scale, developed by Pratto, Sidanius, Stallworth and Malle [93], was used, which is a measure with high reliability (α = .91) and construct validity. The chosen Spanish version was translated by Silván-Ferrero and Bustillos [94], demonstrating good reliability (α = .85) and predictive validity. Our data also showed very satisfactory internal consistency (α = .86). The measure consisted of 16 items and, as in the previous cases, a five-point response scale was used (1 = “Totally disagree”, and 5 = “Totally agree”). Half the items were reverse coded (9, 10, 11, 12, 13, 14, 15, 16).

#### Empathy

The Cognitive and Affective Empathy Scale (TECA, Spanish acronym) developed by López-Pérez, Fernández-Pinto and Abad [95] was used in this study. This gives an overall measure of empathy that consists of 33 items grouped into four factors. The first two factors measure cognitive processes: Perspective Taking (8 items) refers to the intellectual or imaginative capacity to put oneself in the place of the other, while the Emotional Understanding factor (9 items) refers to the capacity to recognise and comprehend the emotional states of others. The two remaining factors measure emotional processes: Empathic Distress (9 items), which refers to negative emotional resonance or the capacity to share the negative emotions of others, while Empathic Joy (7 items) refers to positive emotional resonance. Cronbach's alpha in the norm group was .86 for overall TECA and between .70 and .78 for its four factors. In our data, Cronbach's alpha was .83 for overall TECA. For the 4 components of empathy, Cronbach's alpha was .72 in Perspective Taking, .61 in Emotional Understanding, .75 in Empathic Distress and .70 in Empathic Joy. Given that the reliability of the second component was lower than .70, it was disregarded in the critical analyses, meaning the final score for empathy was calculated using three components; one cognitive (Perspective Taking) and two emotional (Empathic Distress and Empathic Joy). Internal consistency for the 24 items of these 3 factors was also .83. A five-point response scale was used (1 = “Totally disagree”, and 5 = “Totally agree”). Twelve of the items were reverse coded (7, 8, 10, 12, 14, 17, 21, 25, 26, 28, 30 and 32).

#### Prejudice

Prejudice attitudes were measured using the Subtle and Blatant Prejudice Scales developed by Pettigrew and Meertens [27], adapted for use in Spain by Rueda and Navas [96], with the formulation of some items improved by Marisol Navas and her colleagues ([97] [98]) to make the statements easier to understand. She also added to the five original subscales a series of negative emotions whose items were grouped together in a new subscale: Subtle Negative Emotions. As in the study by Navas, Maghrebi immigrants were used as the reference group in the items, as they represent the largest non-European national minority group in Spain. Within this group, the Moroccan faction is most associated by the majority population group with the phenomenon of immigration which, in turn, is linked to stereotypes and prejudice [99]. There were two subscales for the blatant prejudice scale: Threat and Rejection (6 items), which measures overt rejection of the out-group and the perceived threat of appropriation of the in-group's resources; and Intimacy (5 items), which measures rejection of contact and close relationships with the out-group. In turn, the subtle prejudice scale has four subscales: Traditional Values (4 items), which measures defense of the in-group's traditional values; Cultural Differences (7 items), which assesses the exaggerated perception of cultural differences between the in-group and the out-group; Affective Prejudice (2 items), which measures the expression of positive emotions towards the out-group; and Subtle Negative Emotions (5 items), which measures the expression of negative emotions that are not overtly hostile towards the out-group. In the case where Maghrebis were the target group towards which the prejudicial attitudes were assessed, Navas et al. [97] reports reliability coefficients for the different subscales of between .74 and .86 except for Traditional Values, in which the alpha value was insufficient. In our data, the alpha values were: 78 in Threat and Rejection, .82 in Intimacy, .57 in Traditional Values, .74 in Cultural Differences, .71 in Affective Prejudice, and .82 in Subtle Negative Emotions. Just as with Navas et al. [97], the Traditional Values scale was disregarded and the individual scores on the subtle prejudice scale were calculated using the three remaining subscales. To calculate the scores for blatant prejudice, it was possible to use both the Threat and Rejection subscale and the Intimacy subscale. In the end, the internal consistency analysis for the blatant prejudice and the subtle prejudice scales produced alpha values of .84 and .81, respectively. A five-point response scale was used, with a different meaning assigned to the extreme points of the scale depending on the statement or question (1 = “Totally disagree”/“Not angry at all”/“Very dissimilar”/“Never”), and 5 = “Totally agree”/“Very angry”/“Very similar”/“Very frequently”). The last three items of the subscale Intimacy and the seven items of the subscale Cultural Differences were reverse coded.

### Procedure

The participants completed the measures in 4 groups. Each group passed through an IT room, where one of the researchers told the students the major aims of the study, reminded them that the task was completely voluntary and fully anonymous, and asked them to sign a consent form if they agreed to respond to measures. Otherwise, the participants could withdraw from the data collection process. After that, the researcher gave them a link to the platform where the instruments were hosted. Each student first gave their gender and age, then completed the instruments in the order presented in the foregoing section. Upon completion, participants were debriefed and thanked for their time and effort. The responses were stored on the program in an Excel spreadsheet, and the data file was later opened with SPSS software.

## Results

### Statistical analysis

After recoding the items that had been reverse coded in the various measures, basic descriptive and correlation analysis was performed on the variables, and then the hypothetical model reproduced in Fig 1 was tested through path analysis (Amos v.22). As a preliminary step, in all the data distributions we revised the critical ratios of skewness and kurtosis to their standard errors (Amos v.22). Likewise, the multivariate kurtosis was also assessed. For improved levels of normality, the Mahalanobis distance (*d*^*2*^) was used to detect outliers (*p1* < .05).

The maximum likelihood estimation method was selected to perform the confirmatory path analysis, and the model fit was assessed using the following indices: Chi-square to degrees of freedom ratio (*χ*^*2*^*/df*), Comparative Fit Index (CFI), Tucker Lewis Index or Non-Normed Fit Index (TLI or NNFI), Standardized Root Mean Square Residual (SRMR), and Root Mean Square Error of Approximation (RMSEA) and its 90% confidence interval. Although some of the indices are associated with various limitations [100–108], the convergence between them was considered in order to make a decision about the goodness of fit.

### Explanatory dual-process model of blatant and subtle prejudice

In an elaborate model-specification process, the optionality of certain effects unforeseen in the hypothetical model was tested in order to improve the model fit and, simultaneously, make visible the effectiveness of empathy in the framework of the dual process model. Then, the pattern of effects that best fitted the empirical data was determined, obtaining overall satisfactory indices (*X*^*2*^*/df* = 2.55; *CFI* = .971; *NNFI* = .926; *SRMR =* .054; *RMSEA =* .080 [.044 –.117]) in a model with six variables explaining blatant and subtle prejudice: three concerning personality (openness to experience, neuroticism and agreeableness), two concerning attitudes (RWA and SDO), and general empathy. Prior to this, for normalization purposes, Mahalanobis distance was used to identify multivariate outliers, detecting 17 cases in two successive rounds, that were removed. As such, the statistic corresponding to the multivariate kurtosis (Mardia's coefficient) produced a low value (*MK* = -3.30) and, in any case, *c*.*r*. = -2.03, with this standardized index coming in below the 5.00 threshold suggested by Bentler [109] as the maximum acceptable value for presupposing multivariate normality. Therefore, the final sample group consisted of 243 participants: 200 women (82.3%) and 43 men (17.7%).

Table 1 presents the descriptive statistics and Pearson correlations between the eight variables in the model. Twenty-four of the twenty-eight correlations reached statistical significance, in the manner anticipated in the literature. It should be noted that, among the two Big Five factors that are usually very consistently linked to prejudice (openness to experience and agreeableness), openness was correlated negatively with blatant and subtle prejudice more strongly than expected. Nevertheless, the association between the personality trait and blatant prejudice dropped to *r* = -.18, *p* = .006, when controlling for both SDO and RWA, and to *r* = –.23, *p* < .001, when controlling only for RWA; while partial correlations between the trait and subtle prejudice were *r* = -.17, *p* = .007, and *r* = -.18, *p* = .004, respectively. Therefore, correlations were markedly lower when controlling for both ideological attitudes relative to the equivalent bivariate association between openness and both kinds of prejudice, while controlling for RWA alone produced only a slight increase relative to controlling for RWA and SDO, suggesting that the association between openness and prejudice was due largely to share variance with RWA but not with SDO.

**Table 1:** Mean, standard deviation and Pearson product-moment correlations between the variables in the model of blatant and subtle prejudice (N = 243).

Variable	*M*	*SD*	2	3	4	5	6	7	8
1. Openness	3.48	.484	-.130*	.265***	-.641***	-.449***	.334***	-.516***	-.401***
2. Neuroticism	3.00	.664	—	-.239***	.068	.060	.015	.159*	.243***
3. Agreeableness	3.92	.408		—	-.238***	-.344***	.520***	-.263***	-.108
4. RWA	2.32	.377			—	.472***	-.276***	.586***	.427***
5. SDO	1.63	.482				—	-.407***	.474***	.261***
6. General empathy	3.99	.367					—	-.305***	-.243**
7. Blatant prejudice	1.73	.494						—	.549***
8. Subtle prejudice	2.99	.509							—

*p < 0.05

**p < 0.01

***p < 0.001

In the case of the association between agreeableness and blatant prejudice, *r* = -.09, *p* > .05, when controlling for both SDO and RWA, and to *r* = –.12, *p* > .05, when controlling only for SDO; while partial correlations between the trait and subtle prejudice were *r* = .01, *p* > .05, and *r* = -.02, *p* > .05, respectively. Therefore, the weak to moderate correlation between agreeableness and blatant prejudice became negligible when the effect of SDO was partialled out–a result consistent with the dual process model hypothesis, as it was also the substantial reduction of the association between openness and prejudice when RWA was controlled.

**Fig 2:**
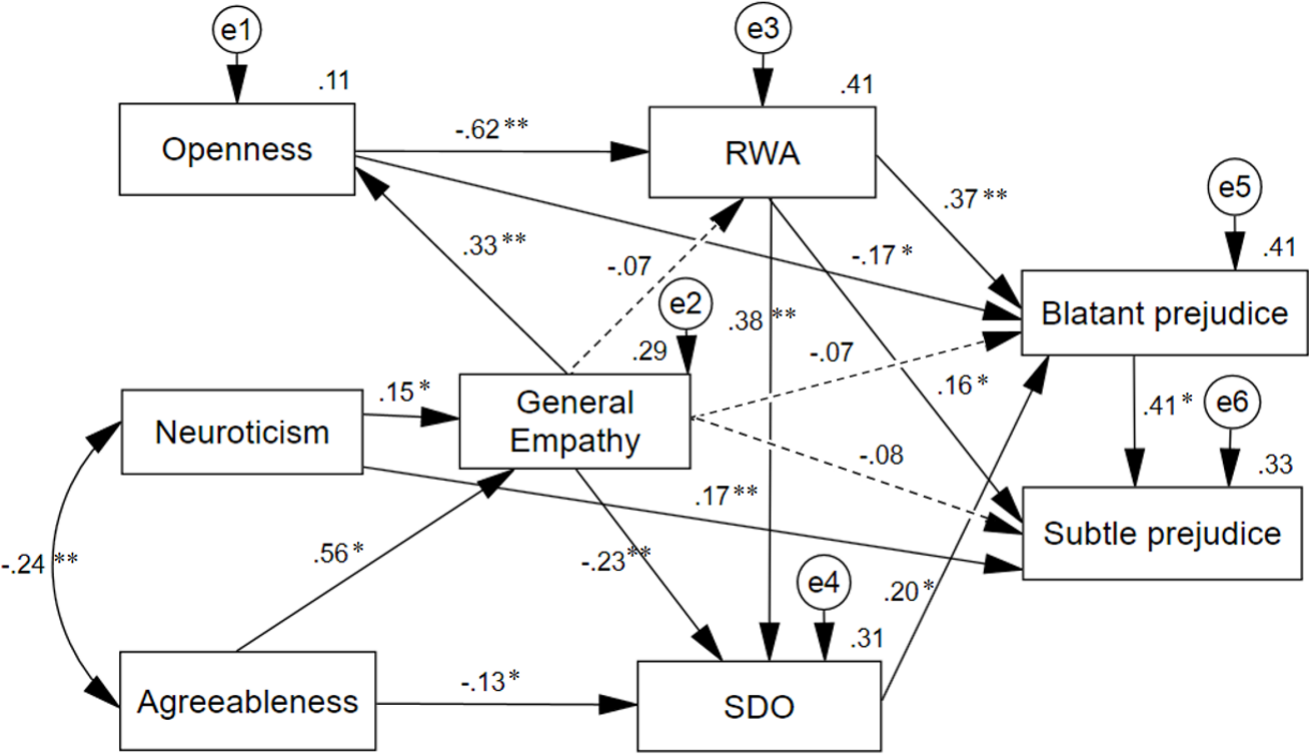
Path analysis of a dual-process model of blatant and subtle prejudice, including general empathy. The standardized regression weights are represented on the arrows, and the squared multiple correlations on the endogenous variables. Dashed arrows indicate expected effects of empathy that did not reach statistical significance. *p < 0.05; **p < 0.01.

The results of the path analysis are presented in Fig 2, confirming the fundamental aspects of the hypothetical model, although a different pattern of effects was observed for both types of prejudice, as well as an unexpected directionality of the association between openness and general empathy. On the one hand, blatant prejudice is explained through the anticipated dual process. That is, openness to experience had a significant impact on right-wing authoritarianism (*β* = -.618, *SE* = .046, *p* = .002) and, in turn, this attitude influenced blatant prejudice (*β* = .367, *SE* = .059, *p* = .007). Agreeableness had an effect on social dominance orientation (*β* = -.134, *SE* = .065, *p* = .045) that, in turn, determined blatant prejudice (*β* = .198, *SE* = .060, *p* = .020). Non-compliance with the model was only observed in the direct effect of openness on blatant prejudice (*β* = -.172, *SE* = .071, *p* = .034), whose addition improved the fit in the model specification process, Δ *χ* ^*2*^ (1) = 6.54, *p* < .05. This effect and the effects corresponding to the dual process of the hypothetical model explained 41% of the variance of blatant prejudice.

The pathway of openness to blatant prejudice through RWA appears dominant in the explanation of blatant prejudice, whose variance would fall to 24% if both predictors were eliminated, while if agreeableness and SDO were removed, the squared multiple correlation would only be slightly reduced in relation to the dual-process model, *R*^*2*^ = .39. This is consistent with bivariate and partial correlations found, and also with the difference between the standardized total effect of openness (*β* = -.444, *SE* = .060, *p* = .003) and that of agreeableness (*β* = -.189, *SE* = .038, *p* = .025) on blatant prejudice, *p* < .05.

The pattern of effects is different, however, for subtle prejudice, whose explanation directly involves one of the hypothetical model pathways—from openness to prejudice through authoritarianism—, in addition to receiving the direct influence of neuroticism (*β* = .171, *SE* = .046, *p* = .004), that improved the fit in the model specification process, Δ *χ* ^*2*^ (1) = 9.10, *p* < .01; as well as the indirect effects of personality, empathy, RWA and SDO through blatant prejudice. Together, these variables explained 33% of the variance of subtle prejudice. No statistically significant difference was observed between the explanatory pathway of openness and RWA on blatant prejudice and the pathway of these same variables on subtle prejudice. Although openness had both a direct and an indirect effect on blatant prejudice, RWA also had both a direct and an indirect effect—through blatant prejudice—on subtle prejudice, meaning that the standardized total effect of openness on blatant prejudice (*β* = -.444, *SE* = .060, *p* = .003) was not statistically higher than the effect on subtle prejudice (*β* = -.280, *SE* = .041, *p* = .007), *p* > .05, nor was the difference between the total effects of RWA on blatant (*β* = .442, *SE* = .056, *p* = .011) and subtle prejudice (*β* = .339, *SE* = .054, *p* = .008), *p* > .05.

Mapping a blatant prejudice effect to subtle prejudice emerged as a better option during the model specification, although the difference between this effect and a two-way influence between both types of prejudice was negligible in terms of fit, Δ *χ* ^*2*^ (1) = 2.507, *p* > .05. The decision was made to preserve the best-fit effect under the Bayes Information Criterion, since the one from subtle to blatant prejudice did not reach statistical significance. The only effect from blatant to subtle prejudice was, however, significant (*β* = .411, *SE* = .061, *p* = .012).

A second notable category of results from the fitted model refers to empathy. This variable had no significant direct effect on prejudice. The standardized regression weights did not reach statistical significance, neither with regard to blatant prejudice (*β* = -.068, *SE* = .058, *p* = .207) nor WITH subtle prejudice (*β* = -.079, *SE* = .059, *p* = .206). However, in the final model, empathy was seen to have a significant indirect effect on blatant prejudice through openness, RWA and SDO (*β* = -.225, *SE* = .046, *p* = .012), and on subtle prejudice via the same mediators and blatant prejudice (*β* = -.164, *SE* = .034, *p* = .012). The total standardized effects of empathy on both types of prejudice were even higher when non-significant direct effects were added (blatant: *β* = -.293, *SE* = .059, *p* = .019; subtle: *β* = -.243, *SE* = .059, *p* = .013). Empathy also appeared to be a mediator between agreeableness and openness, and between neuroticism and openness. On the other hand, agreeableness and neuroticism explained 29% of the variance of empathy, with agreeableness (*β* = .555, *SE* = .051, *p* = .020) having a significantly greater effect than neuroticism (*β* = .147, *SE* = .056, *p* = .013), *p* < .05.

**Fig 3:**
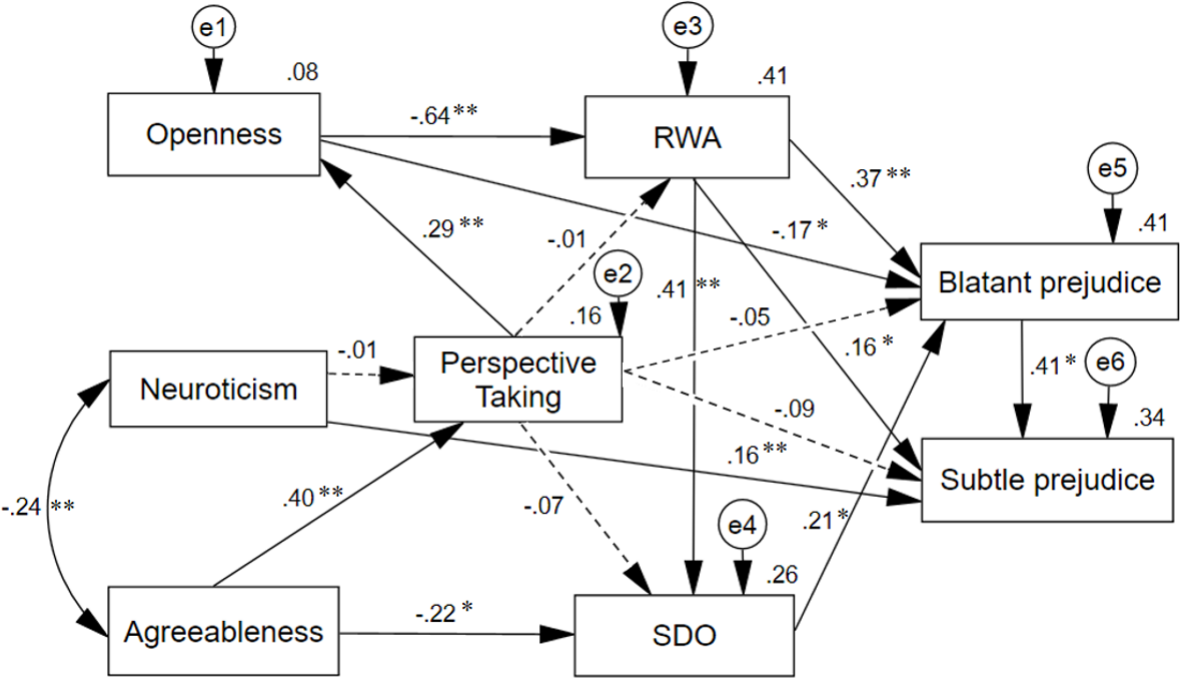
Path analysis of a dual-process model of blatant and subtle prejudice, including perspective taking. The standardized regression weights are represented on the arrows, and the squared multiple correlations on the endogenous variables. Dashed arrows indicate expected effects associated to empathy that did not reach statistical significance. *p < 0.05; **p < 0.01.

**Fig 4:**
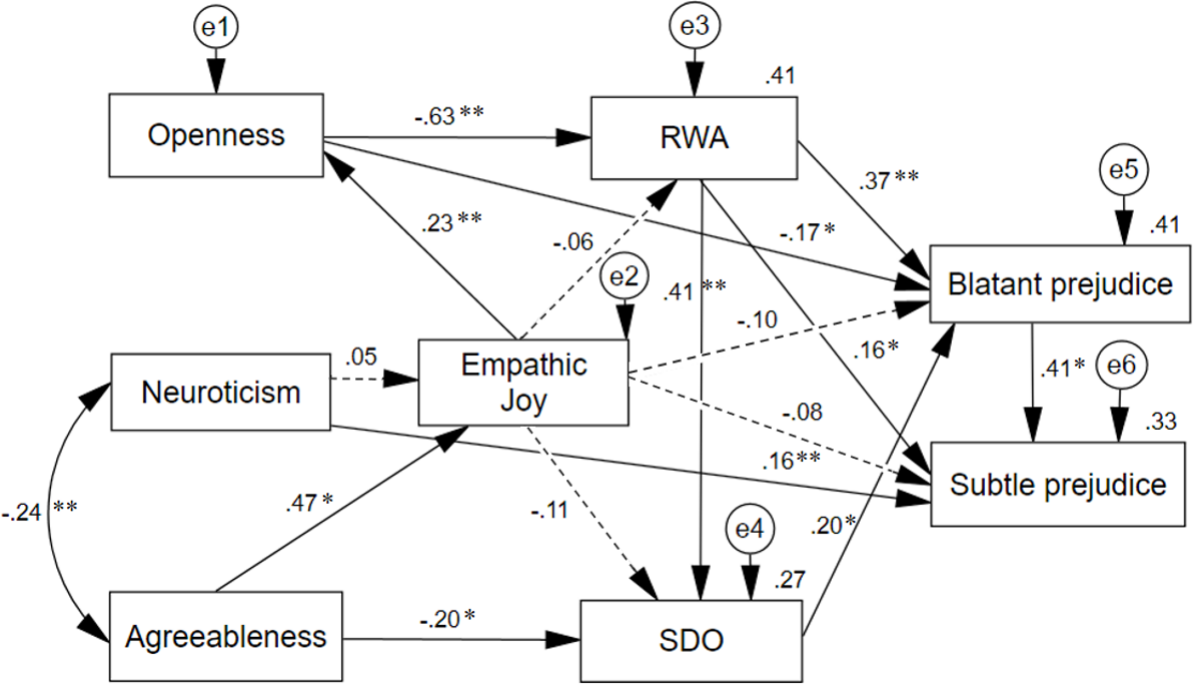
Path analysis of a dual-process model of blatant and subtle prejudice, including empathic joy. The standardized regression weights are represented on the arrows, and the squared multiple correlations on the endogenous variables. Dashed arrows indicate expected effects associated to empathy that did not reach statistical significance. *p < 0.05; **p < 0.01.

Substituting general empathy with each of its components for which reliable data was available (perspective taking, empathic distress, empathic joy), the best model fit was obtained both with perspective taking (*X*^*2*^*/df* = 2.81; *CFI* = .962; *NNFI* = .903; *SRMR =* .060; *RMSEA =* .086 [.051 –.123]) and empathic joy (*X*^*2*^*/df* = 2.48; *CFI* = .972; *NNFI* = .923; *SRMR =* .039; *RMSEA =* .078 [.040 –.118]). As seen in Figs 3 and 4, compared to the model that included general empathy, neuroticism had neither a significant effect on perspective taking nor on empathic joy; and the effect of the empathic dimensions on SDO did not reach significance. With these exceptions, the main pattern of effects remains the same as for the model that included general empathy. Neither perspective taking nor empathic joy had any direct, significant impact on blatant and subtle prejudice, although perspective taking did have an indirect impact on blatant prejudice through openness, RWA and SDO (*β* = -.154, *SE* = .042, *p* = .012), and on subtle prejudice via the same mediators and blatant prejudice (*β* = -.117, *SE* = .033, *p* = .020); as well as empathic joy indirectly impacted on blatant prejudice (*β* = -.150, *SE* = .041, *p* = .018) and on subtle prejudice (*β* = -.136, *SE* = .035, *p* = .009). Total effects of perspective taking and empathic joy increased to *β* > .20 on both types of prejudice. Finally, the explained variance of the two types of prejudice (Figs 3 and 4) was similar to that shown in Fig 1.

## Discussion

On occasion, empathy has been presented as the third big predictor of generalized prejudice, after right-wing authoritarianism and social dominance orientation [20,83]. In the model tested in this study, empathy did not show any type of direct impact on prejudice, but played a relevant role in the mediation between personality, ideological attitudes and prejudice, although it acquired a distinctive character in comparison with explanations of generalized prejudice. To the extent that the pathway from openness to blatant prejudice through right-wing authoritarianism appeared dominant, openness and RWA (and not RWA and SDO, as in the explanation of generalized prejudice) accounted for the most variance, followed by empathy. With regard to subtle prejudice, openness, RWA and blatant prejudice were the three most relevant predictors, followed by empathy. These are the general findings of our study, which marks the first time that empathy has been incorporated into John Duckitt's Dual-Process Cognitive-Motivational Model [9–12].

There are various interpretations attributable to these findings, which will contribute to the development of theories regarding the relationships between personality, ideological attitudes and empathy. The first of these interpretations indicates that the cognitive and affective components of empathy are to some extent distributed in the explanatory model of prejudice, being a variable that correlates consistently with agreeableness, openness, RWA and SDO. Facets of agreeableness, such as altruism or tender-mindedness; or flexibility, unconventionalism and interest in the external world of people scoring highly in openness to experience, are in themselves empathetic ingredients. In particular, agreeableness is the most powerful transcultural predictor of empathy [81]. In turn, components such as authoritarian aggression and conventionalism, or the defense of hierarchy and inequality are associated with the lowest levels of empathetic disposition. When empathy is included in this network of variables, it plays a mediational role, mostly between the two personality traits that are closely linked to it [75, 76, 78, 80, 81], as well as between personality and social dominance–variable found to be predicted by empathy in other studies [83, 85]. In this way, empathy might be properly categorized as a stable disposition with a biological basis that appears very early in human life [110], and that would have the potential to influence other dispositions, as is the case of openness to experience.

Even considering the significant role of empathy shown in our study, two plausible reasons that would have limited its effectiveness should be put forward for discussion. The first relates to the lack of variability in the distribution of general empathy data, with this reduced diversity limiting the correlational and predictive effect sizes in which this variable intervenes. The same applies in reference to SDO. Bäckström and Björklund [83], for example, found empathy to have a much greater effect on SDO than that recorded in our data, being SDO’s mean and standard deviation higher than the ones found in our sample. These statistics for SDO were very low in our data, which might have attenuated its direct effect on blatant prejudice and its indirect one on subtle prejudice, as well as the indirect effects of general empathy on blatant and subtle prejudice.

Another reason that reduces the effect of empathy is connected to the type of instruments used in our study. The TECA scale is an overall measure of empathy that does not consider the perspective taking of and empathic concern for others’ cultures, but only for others’ individual conditions (without associating these conditions to group membership). On the other hand, the Subtle and Blatant Prejudice Scales measured prejudice against an ethnocultural group—Maghrebi immigrants in Spain—. It is possible that empathy towards a generic person, without considering his/her culture, is weakly associated with prejudice towards a specific ethnocultural group. That is, although adopting the perspective of a generic "other" or wanting to share positive emotions with this individual "other" can be judged as ideals, it would hardly predict attitudes towards specific social groups, in the same way that high levels of contextualized empathy might be weakly predictive of generalized prejudice. The meta-analysis performed by Sibley and Duckitt [12] provided some evidence on this type of interpretation. It consistently confirmed that general measures of agreeableness and openness to experience predicted certain sexist and racist prejudices in a weaker way than when predicting generalized prejudice. In this study, although empathy correlated with both blatant and subtle prejudice, the size of the coefficients was very moderate. In this respect, perhaps more contextualised measures of empathy are called for, like those used in multicultural contexts [111]. In fact, the connection between empathy and culture has already created concepts such as cultural empathy [112], ethnocultural empathy [43,113] cultural competence or cross-cultural empathy [43,114,115], leading to the idea that together with general empathy, it is also possible to include understanding and acceptance of the other's culture. This type of empathy correlates moderately with general empathy, but very strongly, in a negative sense, with prejudice, as shown by Albiero and Matricardi [61]. These authors correlated ethnocultural empathy with subtle prejudice and with blatant prejudice, obtaining coefficients of -.75 and -.68, respectively; values that are a far cry from those found in our own study (.24 for subtle prejudice and .30 for blatant prejudice).

Regarding confirmation of the Dual-Process Cognitive-Motivational Model [9–12] , it basically fitted our data in terms of the explanation of blatant prejudice, but also of subtle prejudice through blatant prejudice. The only unexpected direct effects, excluding empathy, were those of openness on blatant prejudice, and of neuroticism on subtle prejudice, which improved the goodness of fit. The model predicts that RWA and SDO anticipate prejudice towards different social groups according to different perceptions related to threat and to competition, respectively [116]. In the case of our study, where the pathway of openness to prejudice through authoritarianism was stronger than that of agreeableness and SDO, the Maghrebi group would be seen fundamentally as a threat to social values and security, rather than a challenge to the status and power of the in-group. That is, prejudice would be influenced more by motives arising from RWA than by those linked to SDO, with the strongest influence coming from values linked to the defense of tradition, order and social cohesion [10,11,86,116,117]. In fact, this interpretation is supported by the measurements obtained in both variables: *M*_*RWA*_ = 2.33 (*DT* = 0.37), *M*_*SDO*_ = 1.63 (*DT* = 0.48), *t* (242) = 24.33, *p* < .001, *d* = 1.62; as well as by the pattern of partial correlations between personality and prejudice relative to bivariate associations. These results, jointly considered, might indicate a dominant socialization of our sample participants in RWA relative to SDO. In fact, the effect of RWA on SDO could also be adequately interpreted within this socialization framework [118], as well as the unusually stronger negative correlation of openness with prejudice, that might be complementary explained by the use of NEO-FFI as a personality measure, and by the collection of data in a sample of undergraduate students (the size of the association between openness and prejudice is above the mean in the vast majority of studies using NEO-FFI or NEO-PI-R, and in undergraduate student relative to adult samples [12]). Additionally, the analysis of possible differences in the association between personality and prejudice across studies due to prejudice measures would be valuable, since may be the use of the Subtle and Blatant Prejudice Scales [27] biased upward the correlation between openness and prejudice relative, for example, to McConahay's Modern Racism Scale [119], a more frequently used instrument. Meanwhile, the type of perception associated with the reference group, dominated by openness and RWA, would also have contributed to weakening empathy's effect on blatant prejudice through SDO.

In turn, the explanation of subtle prejudice only overlaps with the explanation of blatant prejudice, and this supports confirmation of the difference between these constructs [27,28], despite the notable connection between them [4,26,29,120] that our data also corroborate (*r* = .549, *p* < .001). In other words, unlike studies that have not managed to identify the differentiated nature of these two types of prejudice [121,122], our findings confirm a degree of overlap in the model pattern (importance of the pathway from openness to the two types of prejudice through authoritarianism), while still maintaining the distinction between these constructs: the explanation of blatant prejudice also involves a direct effect from SDO, in addition to the direct effect of openness; while neuroticism has a direct impact on subtle prejudice, which is also influenced by RWA and SDO through blatant prejudice. These differential predictors and paths can therefore be added to the variables already detected in the literature, such as contact, which predicts blatant prejudice to a greater degree than subtle prejudice [123]. On the other hand, the greater relevance of SDO in blatant prejudice compared to subtle prejudice has already been highlighted by Passini and Morselli [29], who detected the direct effect of dominance on classic prejudice, but not on subtle prejudice. Likewise, in a study on similar constructs, Kteily, Bruneau, Waytz and Cotterill [124] showed that support of hierarchy is more closely linked to blatant dehumanization than to subtle dehumanization. This set of effects is congruent with and gives consistency to the pattern found in this study.

The percentage of explained variance of prejudice by personality, attitudes and general empathy, which is limited (41% in blatant and 33% in subtle), should also be mentioned. In other studies, this percentage has fluctuated between 34% and 70%, highlighting the power of prediction of RWA and SDO [2,20,125,126], thus the percentage in our study sits at the lower end of this range. It is probable that the homogenous sociodemographic profile of the participants—young students from Cordoba on education courses, the vast majority being female—may have affected the means and variability of their scores in the measured variables. In other words, future teachers and social educators are expected to score low in neuroticism, RWA, SDO and prejudice, and high in openness and agreeableness. Likewise, the variances of the corresponding distributions are also expected to be low. These predictions, roughly confirmed in our study, naturally limit the effect sizes, as already noted in relation to empathy.

Together with the potential use of more diverse sample groups, a second option that in future studies could contribute to broadening the explained variance is based on considering the facets or dimensions of the personality and attitudinal constructs. Some studies have already been very effective in this respect [23,24,127–129]. In our research, however, the confirmatory analyses were referenced to global constructs in order to facilitate comparability with the majority of the previous literature on the topic.

Finally, the use of intercultural empathy instruments, as already mentioned, could increase the percentage of explained variance of prejudice, in the manner anticipated by the “Big Three” model [20,83], included in the Dual-Process Cognitive-Motivational Model that has been confirmed in this study.

In conclusion, the results of the study partially confirmed the first main hypothesis; namely, Duckitt’s dual process model was clearly identifiable after introducing empathy, being dominant one of the two pathways predicting prejudice (openness to blatant and subtle prejudice through RWA). Within this prevailing motivational orientation, empathy played a significant role between personality traits, and between them and ideological attitudes, ultimately impacting on both modalities of prejudice. Therefore, and although it was not found to have a direct effect on prejudice, empathy could be thought of as a relevant pillar of prejudice when perceiving specific groups as a threat to social values and security. Nonetheless, the issue is still very much an open question that needs to be addressed in future studies by using different samples, instruments, and target groups. For example, it would be of great interest to test if empathy is still the third main predictor of prejudice when perceiving social groups considered as a challenge to the status and power of the in-group.

Secondly, the pattern of effects was different for blatant and subtle prejudice in a way consistent with the second main hypothesis. Mostly, blatant prejudice received the effects predicted by the model (direct effects of empathy being an exception), while subtle prejudice was impacted by personality, empathy and ideology in a weaker and indirect way, mainly through blatant prejudice. A deeper analysis will be needed to draw contrasted, consistent conclusions about these differential effects, as well as about the overlapping nature of both types of prejudice. In this task, the use of other measures of old-fashion and contemporary prejudice might be relevant.

## Supporting information

S1 Dataset Data file corresponding to the final sample (N = 243) (item scores and scale/subscale scores). (XLSX)
